# Clinical efficacy of amrubicin in patients with small cell lung cancer relapse after first‐line treatment including immune checkpoint inhibitors: A retrospective multicenter study (TOPGAN 2021‐01)

**DOI:** 10.1111/1759-7714.14729

**Published:** 2022-11-21

**Authors:** Shinya Uematsu, Satoru Kitazono, Hisashi Tanaka, Ryota Saito, Yosuke Kawashima, Fumiyoshi Ohyanagi, Takehiro Tozuka, Tsugitomi Ryosuke, Toshio Sakatani, Atsushi Horiike, Takahiro Yoshizawa, Masafumi Saiki, Yuichi Tambo, Junji Koyama, Masaki Kanazu, Keita Kudo, Yuko Tsuchiya‐Kawano, Noriko Yanagitani, Makoto Nishio

**Affiliations:** ^1^ Department of Respiratory Medicine Osaka Red Cross Hospital Osaka Japan; ^2^ Department of Thoracic Medical Oncology The Cancer Institute Hospital, Japanese Foundation for Cancer Research Tokyo Japan; ^3^ Department of Respiratory Medicine Hirosaki University Graduate School of Medicine Hirosaki Japan; ^4^ Department of Respiratory Medicine Tohoku University Hospital Sendai Japan; ^5^ Department of Pulmonary Medicine Sendai Kousei Hospital Sendai Japan; ^6^ Department of Respiratory Medicine Saitama Cancer Center Saitama Japan; ^7^ Department of Pulmonary Medicine and Oncology Graduate School of Medicine, Nippon Medical School Tokyo Japan; ^8^ Division of Respiratory NTT Medical Center Tokyo Japan; ^9^ Division of Medical Oncology, Department of Medicine Showa University School of Medicine Tokyo Japan; ^10^ Department of Respiratory Medicine Toho University School of Medicine Tokyo Japan; ^11^ Department of Respiratory Medicine Graduate School of Medicine University of Yamanashi Yamanashi Japan; ^12^ Department of Respiratory Medicine Kanazawa University Kanazawa Japan; ^13^ Department of Respiratory Medicine Nagoya University Graduate School of Medicine Nagoya Japan; ^14^ Department of Thoracic Oncology National Hospital Organization Osaka Toneyama Medical Center Osaka Japan; ^15^ Department of Medical Oncology and Respiratory Medicine National Hospital Organization Osaka Minami Medical Center Osaka Japan; ^16^ Department of Respiratory Medicine Kitakyushu Municipal Medical Center Kitakyushu Japan

**Keywords:** amrubicin, atezolizumab, durvalumab, immune checkpoint inhibitor, small cell lung cancer

## Abstract

**Background:**

The therapeutic efficacy of cytotoxic anticancer drugs has been reported to be enhanced after immune checkpoint inhibitors (ICI) in non–small cell lung cancer; however, it is unclear whether the same is applicable for small cell lung cancer (SCLC). We evaluated the efficacy of second‐line amrubicin (AMR) following first‐line platinum‐based chemotherapy and ICI combination therapy (chemo‐ICI) in SCLC.

**Patients and Methods:**

We retrospectively enrolled consecutive patients with SCLC treated with AMR as a second‐line following chemo‐ICI as first‐line between July 2019 and April 2021 from 16 institutions throughout Japan. We investigated the therapeutic effectiveness, safety, and efficacy‐enhancing variables of AMR.

**Results:**

Overall, 89 patients treated with AMR after first‐line chemo‐ICI were analyzed. The overall response rate (ORR) was 29.2% (95% confidence intervals [CI], 20.1–39.8) and median PFS (m PFS) was 2.99 months (95% CI, 2.27–3.65). Patients who relapsed more than 90 days after receiving first‐line platinum combination therapy (sensitive relapse) exhibited greater ORR (58.3% vs. 24.7%, *p* = 0.035) and m PFS (5.03 vs. 2.56 months, *p* = 0.019) than patients who relapsed in <90 days (refractory relapse). Grade 3 or higher adverse events were mainly hematological toxicity.

**Conclusions:**

Our study suggested that the therapeutic effect of AMR was not enhanced after ICI on SCLC. However, AMR may be effective in cases of sensitive relapse after chemo‐ICI. There was no increase in severe toxicity associated with AMR after ICI.

## Graphical abstract

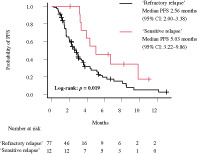

## INTRODUCTION

Small cell lung cancer (SCLC) accounts for ~15% of lung cancers, and two‐thirds of patients with SCLC are classified with extensive disease (ED) at the time of diagnosis.^1^ Over the past 30 years, the standard of care for first‐line treatment of ED‐SCLC has been platinum‐based chemotherapy; despite its high response rate, almost all patients relapse.^2^, ^3^ The addition of immune checkpoint inhibitors (ICI) to platinum‐based chemotherapy in the first‐line treatment of ED‐SCLC has recently been demonstrated to prolong survival and has been introduced into clinical practice. However, most patients still relapse, and the median overall survival (OS) is ~1 year.^4^, ^5^

In ED‐SCLC, the efficacy of second‐line therapy depends on the time between the completion of first‐line platinum‐based chemotherapy and recurrence. Sensitive relapse is defined as the relapse that occurs 90 days or more from the completion of first‐line treatment, whereas refractory relapse is defined as the relapse that occurs within 90 days of treatment.^6^, ^7^ Sensitive relapse is more likely to respond to second‐line therapy than does refractory relapse.^8^ Although topotecan is the standard treatment for sensitive relapse in Europe and the United State,^9^, ^10^ amrubicin (AMR) has been reported to have an objective response rate (ORR) of 61% and a one‐year survival rate of 51% in the Japanese population,^11^ making AMR a standard second‐line treatment. Although no standard treatment for refractory relapse has been established, AMR has been reported to be effective in the Japanese population with a response rate of 33% and a 1‐year survival rate of 36%.^12^ Therefore, it is used in daily clinical practice. Based on these results, AMR is often used as second‐line therapy for relapsed ED‐SCLC following platinum‐based chemotherapy in Japan, both for sensitive and refractory relapse.

This evidence for second‐line therapy was reported before using ICI for first‐line treatment of ED‐SCLC. ICI has only recently been introduced for the treatment of SCLC, therefore, evidence is limited with respect to the therapeutic efficacy of second‐line regimens after first‐line intervention using ICI‐containing regimens. ICI has previously been introduced into clinical practice for non–small cell lung cancer (NSCLC), and several studies have reported that the treatment with ICI was followed by an increase in the therapeutic effect of subsequent chemotherapy in NSCLC.^13^, ^14^

We hypothesized that ICI treatment would increase the efficacy of subsequent chemotherapy not only in NSCLC, but also in SCLC. In this study, we investigated the therapeutic efficacy of AMR, the standard second‐line therapy in Japan, in patients who received ICI as first‐line therapy for ED‐SCLC.

## MATERIALS AND METHODS

### Study design

This was a multicenter, retrospective cohort study conducted at 16 hospitals participating in the TOPGAN group in Japan. The study was approved by the institutional review board of Osaka Red Cross Hospital (approval number: J‐0229) and other participating hospitals. Written informed consent was not obtained owing to the retrospective nature of this study.

### Data collection

We reviewed the clinical data for each patient extracted from the medical records. Patients clinically diagnosed with ED‐SCLC or those relapsed with limited disease SCLC after chemoradiotherapy were enrolled, if they were treated with AMR as a second‐line treatment between July 2019 and April 2021 following platinum‐based chemotherapy and ICI combination therapy (chemo‐ICI) as the first‐line treatment. Patients who relapsed after the first‐line chemo‐ICI and received platinum‐based chemotherapy as a re‐challenge prior to AMR were excluded. First‐line chemo‐ICI was a combination of platinum, etoposide, and ICI (atezolizumab or durvalumab) up to four courses. Patients were started on AMR as second‐line therapy after disease progression as described above. AMR was administered at a dose of 30 to 40 mg/m^2^/day for three consecutive days, every 21 days or longer, until disease progression or intolerable toxicity.

Clinical characteristics of patients before starting AMR were obtained from medical records. Patients with recurrence after first‐line chemo‐ICI were classified as sensitive relapse or refractory relapse based on the conventional criteria.^15^ We defined sensitive relapse as the time from the completion of platinum based chemotherapy to recurrence of 90 days or more and refractory relapse as the time from the completion of platinum based chemotherapy to recurrence of <90 days. The primary outcome of this study was the ORR to second‐line AMR for relapse after first‐line chemo‐ICI. The secondary outcomes included disease control rate (DCR), progression‐free survival (PFS), and safety of second‐line AMR therapy. Disease control was assessed post hoc by investigators and defined as having two or more scans with RECIST version 1.1‐defined stable disease, once documented stable disease followed by a non‐confirmed partial response, or a confirmed complete or partial response.^16^ PFS was calculated as the interval between initiating AMR and the date of clinical or radiographic disease progression or any‐cause death. Data were censored from the last date when patient survival was confirmed if the PFS event was unknown. We analyzed the difference in treatment effect between sensitive relapse and refractory relapse according to the classification already described. We also collected adverse events (AEs) and these were graded according to the common Terminology Criteria for Adverse Events (version 5.0).^17^ The cutoff date was June 30, 2021.

### Evaluation and statistical analysis

We performed all statistical analyses using EZR (ver.1.52) (Saitama Medical Center, Jichi Medical University).^18^ Baseline characteristics were compared between sensitive relapse and refractory relapse using the Fisher's exact test in the case of categorical variables or Mann–Whitney *U* test for continuous or ordinal variables. PFS were calculated using Kaplan–Meier analyses, and the log‐rank test was used to compare survival rates between the two groups. Hazard ratios (HR) and associated 95% confidence intervals (CI) were calculated using a Cox proportional hazards model. Univariate and multivariate analyses with Cox proportional hazard analysis were used to evaluate prognostic factors. Based on previous reports on predictors of treatment response in recurrent SCLC^15^ and prognostic factors for chemotherapy after ICI in NSCLC,^19^ we built a model for multivariate analysis using age (≥75), performance status (PS),^2^, ^3^ and sensitivity to first‐line chemo‐ICI (sensitive relapse). Differences were assumed to be significant at *p*‐value of <0.05.

## RESULTS

### Patient characteristics

We included 89 patients treated with AMR after first line chemo‐ICI from 16 participating institutions of the TOPGAN group. Baseline characteristics of these patients are shown in Table 1. Of the 89 patients, 12 (13.5%) were sensitive relapse, and 77 (86.5%) were refractory relapse. The majority of patients were male (83.1%), had a history of smoking, or exhibited good PS. Of the 89 patients, 34 (38.2%) had brain metastases before AMR started, and 18 (52.9%) received whole‐brain radiation or gamma knife therapies. The starting dose of AMR was <35 mm/m^2^ in over half of all patients. There were no significant differences in patient characteristics between the two groups of sensitive relapse and refractory relapse, including brain metastases and the presence or absence of local treatment for brain metastases.

**TABLE 1:** Baseline characteristics in patients who received AMR therapy (*n* = 89)

	Total *n* = 89	Refractory relapse^a^ *n* = 77	Sensitive relapse^b^ *n* = 12	*p*‐value
Age, median (range), years	70 (39–82)	71 (39–82)	69.5 (57–80)	0.75
Gender, *n* (%)				
Female	15 (16.9)	12 (15.6)	3 (25.0)	0.42
Male	74 (83.1)	65 (84.4)	9 (75.0)	
PS, *n* (%)				
0–1	78 (87.6)	67 (87.0)	11 (91.7)	1.00
2–3	11 (12.4)	10 (13.0)	1 (8.3)	
Smoking history, *n* (%)				
Current or former	85 (95.5)	74 (96.1)	11 (91.7)	0.45
Never	4 (4.5)	3 (3.9)	1 (8.3)	
Stage, *n* (%)				
III or recurrent	15 (16.9)	11 (14.3)	4 (33.3)	0.11
IV	74 (83.1)	66 (85.7)	8 (66.7)	
Brain metastases, *n* (%)				
Present	34 (38.2)	29 (37.7)	5 (41.7)	1.00
Absent	55 (61.8)	48 (62.3)	7 (58.3)	
Previous ICI as 1st line, *n* (%)				
Durvalumab	6 (6.7)	6 (7.8)	0 (0.0)	1.00
Atezolizumab	83 (93.3)	71 (92.2)	12 (100.0)	
Starting dose of AMR (mg/mm^2^)				
40	33 (37.1)	29 (37.7)	4 (33.3)	0.68
35	45 (50.6)	39 (50.6)	6 (50.0)	
30	11 (12.4)	9 (11.7)	2 (16.7)	

*Note*: Data are presented as *n* (%) unless otherwise indicated.

Abbreviations: AMR, amrubicin; ICI, immune checkpoint inhibitor; PS, performance status.

^a^ Time from the end of platinum‐based chemotherapy to recurrence of fewer than 90 days.

^b^ Time from the end of platinum‐based chemotherapy to recurrence of 90 days or more.

### Efficacy

**FIGURE 1:**
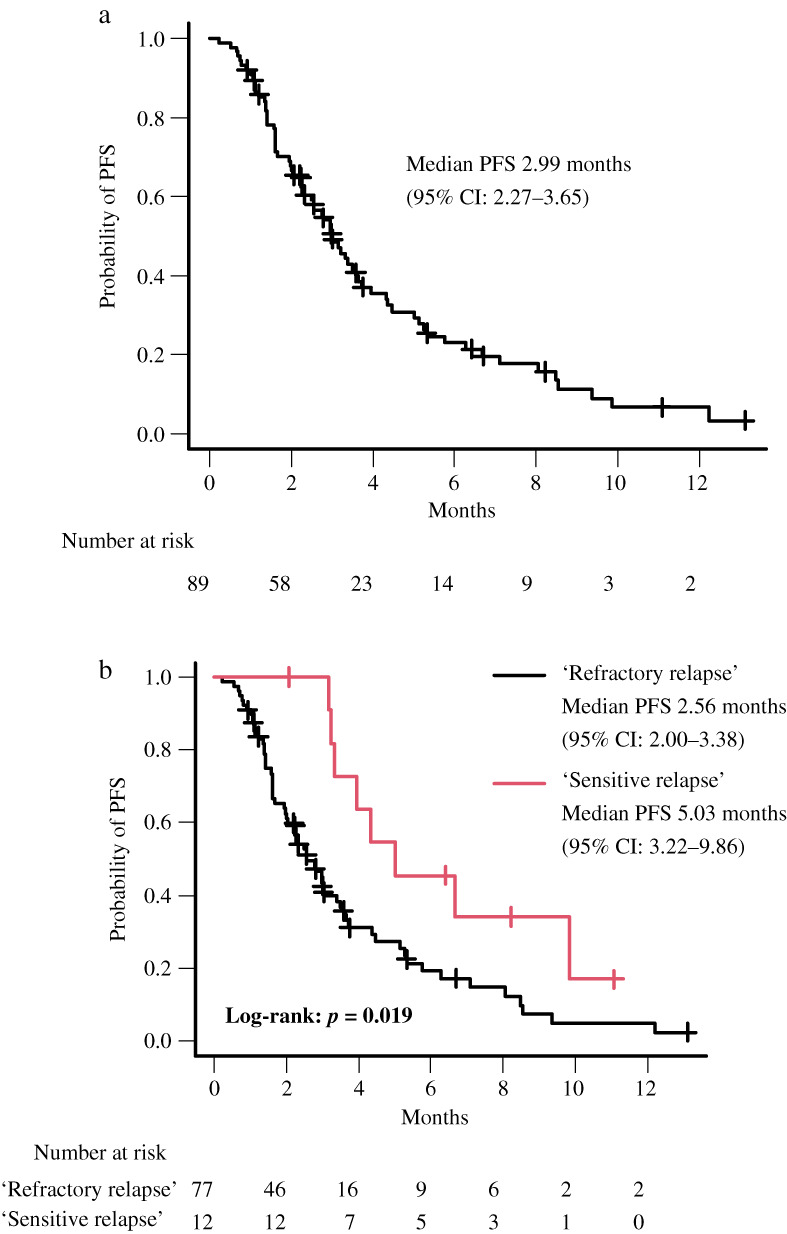
Kaplan–Meier estimates of progression free survival (PFS) from the initiation of second line amrubicin in 89 patients (a) and patients with sensitive relapse * and refractory relapse † (b). *The time from the end of platinum‐based chemotherapy to recurrence in fewer than 90 days. †The time from the end of platinum‐based chemotherapy to recurrence in 90 days or more

The response to AMR is shown in Table 2. The ORR, primary endpoint, was 29.2% (95% CI, 20.1–39.8). The DCR was 59.6% (95% CI, 48.6–69.8). The ORR was significantly higher in patients with sensitive relapse, 58.3%, than that the 24.7% ORR in patients with refractory relapse (*p* = 0.035). Similarly, patients with sensitive relapse had significantly higher DCR than those with refractory relapse (91.7% vs. 54.5%, *p* = 0.024). The median PFS (m PFS) was 2.99 months (95% CI, 2.27–3.65) (Figure 1(a)). Because of the large number of missing values, the median OS was not calculated. PFS of each subgroup is shown in Table 3. There were no differences in PFS of AMR by age (<75), gender, smoking history, clinical stage, presence of brain metastases, or type of ICI used in the first‐line treatment. In univariate analysis, patients with good PS (0–1) tended to have longer PFS than with PS^2^, ^3^ (3.32 vs. 1.94 months, *p* = 0.060), and PFS was significantly longer in the sensitive relapse group than in the refractory relapse group (5.03 vs. 2.56 months, *p* = 0.019) (Figure 1(b)). Multivariate analysis showed that sensitivity to first‐line chemo‐ICI was an independent prognostic factor for PFS (HR,  0.43; 95% CI, 0.20–0.91, *p* = 0.03) (Table 4).

**TABLE 2:** Best overall response rate in patients who received AMR therapy

	Overall *n* = 89	Refractory relapse^a^ *n* = 77	Sensitive relapse^b^ *n* = 12	*p*‐value
Response				
PR, *n* (%)	26 (29.2)	19 (24.7)	7 (58.3)	
SD, *n* (%)	27 (30.3)	23 (29.9)	4 (33.3)	
PD, *n* (%)	32 (36.0)	31 (40.3)	1 (8.3)	
NE, *n* (%)	4 (4.5)	4 (5.2)	0 (0.0)	
ORR (%)	29.2	24.7	58.3	0.035
DCR (%)	59.6	54.5	91.7	0.024

Abbreviations: AMR, amrubicin; DCR, disease control rate; NE, not evaluable; ORR, objective response rate; PD, progressive disease; PR, partial response; SD, stable disease.

^a^ Time from the end of platinum‐based chemotherapy to recurrence of fewer than 90 days.

^b^ Time from the end of platinum‐based chemotherapy to recurrence of 90 days or more.

**TABLE 3:** Progression free survival of each subgroup

	No.	Median PFS (95% CI)	*p*‐value
Age, years			
<75	66	2.99	0.67
≥75	23	3.94	
Gender			
Female	15	5.26	0.18
Male	74	2.96	
PS			
0–1	78	3.32	0.06
2–3	11	1.94	
Smoking history			
Current or former	85	2.99	0.65
Never	4	3.66	
Stage			
III or recurrent	15	2.27	0.40
IV	74	3.02	
Brain metastases			
Present	34	3.32	0.74
Absent	55	2.99	
Sensitivity to 1st line			
Platinum‐based chemotherapy^a^			
Refractory relapse^b^	77	2.56	0.02
Sensitive relapse^c^	12	5.03	
Previous ICI as 1st line			
Durvalumab	6	2.27	0.97
Atezolizumab	83	3.02	

Abbreviations: ICI, immune checkpoint inhibitor; PFS, progression free survival; PS, performance status.

^a^ Time from the completion of platinum‐based chemotherapy to recurrence.

^b^ Time from the end of platinum‐based chemotherapy to recurrence of fewer than 90 days.

^c^ Time from the end of platinum‐based chemotherapy to recurrence of 90 days or more.

**TABLE 4:** Multivariate Cox proportional hazards model for PFS

	Multivariate	
	PFS, hazard ratio (95% CI)	*p*‐value
Age, years (≥75)	1.00 (0.57–1.79)	0.98
PS (2, 3)	1.87 (0.92–3.82)	0.09
Sensitivity to 1st line	0.43 (0.20–0.91)	0.03
Platinum‐based chemotherapy^a^		
(Sensitive relapse^b^)		

Abbreviations: CI, confidence interval; PFS, progression free survival; PS, performance status.

^a^ Time from the completion of platinum‐based chemotherapy to recurrence.

^b^ Time from the end of platinum‐based chemotherapy to recurrence of 90 days or more.

### Safety

Table 5 summarizes AEs of second‐line AMR. The most common Grade 3 to 5 AEs were hematological toxicities, such as neutropenia (43.8%), leukopenia (32.6%), thrombocytopenia (15.7%), anemia (14.6%), and febrile neutropenia (13.5%). Grade 3 or higher enteritis was infrequent, occurring in two patients (2.2%), however, treatment‐related death because of enteritis was observed in one patient (1.1%). Drug‐induced pneumonitis was observed in six patients (6.7%) during AMR treatment, but all patients were grade 3 and no deaths occurred.

**TABLE 5:** Toxicity (≥grade 3) in patients treated with amrubicin (CTCAE v5.0)

	No. (%)	
Adverse events	≥Grade 3	Grade 5
Leukopenia	29 (32.6)	0
Anemia	13 (14.6)	0
Thrombocytopenia	14 (15.7)	0
Neutropenia	39 (43.8)	0
Febrile neutropenia	12 (13.5)	0
Elevated AST/ALT level	1 (1.1)	0
Hyponatremia	4 (4.5)	0
Infection	2 (2.2)	0
Pneumonitis	6 (6.7)	0
Nausea	1 (1.1)	0
Anorexia	2 (2.2)	0
Colitis	2 (2.2)	1 (1.1)
Cardiac disorder^a^	1 (1.1)	0

Abbreviations: AST, aspartate aminotransferase; ALT, alanine aminotransferase.

^a^ Takotsubo cardiomyopathy.

## DISCUSSION

In this study, we assessed the efficacy and safety of AMR after first‐line chemo‐ICI in SCLC patients. Our multicenter retrospective study, which included 89 patients, demonstrated that the therapeutic effect of second‐line AMR after chemo‐ICI was comparable to previously reported data.^12^ Furthermore, our results suggest that sensitivity to first‐line chemo‐ICI was a significant prognostic factor in patients with SCLC receiving second‐line AMR. Concerning safety, the main toxicity of AMR was hematologic, as previously reported, although the frequency of pneumonitis was slightly higher.

The ORR (29.2%) and m PFS (3.0 months) observed in our study were similar to the ORR (32.9%) and m PFS (3.5 months) of a previous prospective study conducted in Japan.^12^ In this study, ORR and PFS were significantly higher in patients who relapsed more than 90 days after the completion of first‐line platinum‐based chemotherapy (sensitive relapse) than in patients who relapsed <90 days (refractory relapse). These results demonstrated the same trend as those before ICI was introduced in the first‐line therapy of SCLC.^8^, ^20^ Although it has been suggested that the therapeutic efficacy of cytotoxic anticancer drugs is enhanced after ICI in NSCLC.^13^, ^14^, ^19^ The results did not suggest an impact of ICI on post‐treatment, as reported for NSCLC. In NSCLC, it has been reported that programmed cell death protein 1 (PD‐1) antibodies promote T cell proliferation and cytokine production,^21^ and activated CD4^+^ T cells enhance the efficacy of cytotoxic anticancer drugs both in vivo and in vitro.^22^ In contrast, although tumor mutational burden (TMB) is high in SCLC, the lack of antigen presentation, immunosuppressive pattern in the stroma, and low expression of PD‐L1 make the lower immunogenic T‐cell profile of SCLC less likely to be as responsive as NSCLC.^23^ Several clinical trials have also shown that immune checkpoints are less effective in SCLC than in NSCLC, which may be related to these backgrounds.^24^, ^25^ Although there are some cases of high immune activity where ICI is effective, the unexpected lower efficacy of AMR after ICI in our results could be because of the lower immune activity in SCLC. From another perspective, AMR used as a second‐line treatment after a first‐line treatment regimen containing etoposide was used in the present study, and this may explain why AMR was less effective. Both etoposide and AMR have the distinction of being topoisomerase II inhibitors. In preclinical studies, topoisomerase II inhibitors have been reported to induce topoisomerase II downregulation and topoisomerase I upregulation.^26^, ^27^, ^28^, ^29^ In a phase II study conducted in Japan, the efficacy of AMR was reported to be lower in patients who received pretreatment with etoposide than in those who received irinotecan, a topoisomerase I inhibitor.^12^ Although the effect of AMR after ICI treatment for SCLC was not as good as expected, our study suggested that AMR was significantly more effective in patients who showed sensitive relapse to first‐line ICI‐containing regimens. Recently, a systematic review also suggests platinum‐doublet regimens may be more effective than AMR or topotecan, the current standard of care for second‐line therapy, especially for sensitive relapse.^30^ A randomized phase III trial also reported that carboplatin plus etoposide significantly prolonged PFS compared with topotecan monotherapy for sensitive relapse (4.7 vs 2.7 months).^31^ PFS of AMR for sensitive relapse in our study was 5.0 months, which may be equivalent to the rechallenge of carboplatin plus etoposide. As per these reports, rechallenge with platinum combination therapy is considered one of the second‐line treatment options for sensitive relapse; however, the results of this study suggest that AMR remains a valid second‐line treatment option after ICI.

In terms of safety, the toxicity of AMR after ICI was comparable to reports before the advent of ICI.^12^ The most common grade 3 or higher AEs were hematological toxicities, which were caused by AMR rather than ICI. The frequency of grade 3 or higher pneumonitis in this study was 6.7%, higher than the previously reported 3.7%,^12^ suggesting the possibility of immune‐related adverse events (irAE) because of ICI in addition to drug failure because of AMR. It was suggested that AMR administration after ICI may increase the frequency of pneumonitis, but all six patients in this study recovered with only corticosteroid administration, and none of them reached grade 5. In addition to pneumonitis, we observed two cases of enteritis, including one grade 5 case, as non‐hematologic toxicities of grade 3 or higher, which was more frequent than previously reported.^12^ In the grade 5 case, pathology specimens from an emergency surgery performed for enteritis ruled out the possibility of irAE. For the other case, although it is difficult to rule out the possibility of irAE, the attending physician judged enteritis to be AMR toxicity based on the clinical course.

## CONCLUSION

The present study has some limitations. First, because this was a retrospective study, the timing of tumor imaging evaluation was not specified, which may have affected PFS. However, the disease progression of SCLC is rapid and often accompanied by fluctuations in tumor markers; therefore, we believe that many patients could have been evaluated appropriately under clinical practice. Second, although the sample size is small, ICI has only been introduced recently in the treatment of SCLC. Hence, under these circumstances, 89 cases were enrolled in this study from 16 centers throughout Japan, which is considered to be a relatively large number of cases.

In conclusion, AMR appears effective for SCLC that relapsed after first‐line treatment including ICI. Our results suggest that pre‐treatment with ICI does not enhance the therapeutic effect of AMR, but it may be a more effective treatment option for patients who respond well to first‐line therapy. For AEs, the possibility of an increased frequency of pneumonitis was suggested; however, the response to corticosteroid therapy was good.

## AUTHOR CONTRIBUTIONS

Shinya Uematsu: Conceptualization, Methodology, Formal analysis, Investigation, Resources, Data curation, Writing – original draft, Writing – review and editing, Visualization and Project administration. Satoru Kitazono: Conceptualization, Methodology, Investigation, Resources, Data curation, Writing – review and editing, Supervision and Project administration. Hisashi Tanaka, Ryota Saito, Yosuke Kawashima, Fumiyoshi Ohyanagi, Takehiro Tozuka, Tsugitomi Ryosuke, Toshio Sakatani, Atsushi Horiike, Takahiro Yoshizawa, Masafumi Saiki, Yuichi Tambo, Junji Koyama, Masaki Kanazu, Keita Kudo, and Yuko Tsuchiya‐Kawano: Investigation, Resources, Data curation and Writing – review and editing. Noriko Yanagitani and Makoto Nishio: Writing – review and editing, Supervision and Project administration.

## DISCLOSURE STATEMENT

Shinya Uematsu report receiving personal fees from AstraZeneca KK, Chugai Pharmaceutical Co. Ltd., NIPPON KAYAKU, Novartis Pharma KK, Eli Lilly Japan KK, Pfizer Japan, Kyowa Kirin, Takeda Pharmaceutical Company, and Ono Pharmaceutical Co. Ltd., outside the submitted work. Satoru Kitazono report AstraZeneca KK, Chugai Pharmaceutical Co. Ltd., Pfizer Japan, and Ono Pharmaceutical Co. Ltd., outside the submitted work. Hisashi Tanaka report receiving personal fees from AstraZeneca KK, Chugai Pharmaceutical Co. Ltd., Pfizer, Ono Pharmaceutical Co. Ltd, Bristol Myers Squibb and Boehringer‐Ingelheim Japan, outside the submitted work. Yosuke Kawashima report receiving personal fees from AstraZeneca KK, Chugai Pharmaceutical Co. Ltd., Eli Lilly Japan KK, and Taiho Pharmaceutical, outside the submitted work. Fumiyoshi Ohyanagi report receiving personal fees from AstraZeneca KK, Chugai Pharmaceutical Co. Ltd., Pfizer, Ono Pharmaceutical Co. Ltd, Bristol Myers Squibb, Novartis Pharma KK, Eli Lilly Japan KK and Merck Sharp and Dohme, outside the submitted work. Atsushi Horiike report receiving grants from Chugai Pharmaceutical Co. Ltd., BeiGene, Merck Sharp and Dohme, Daiichi Sankyo and NIPPON KAYAKU, and receiving personal fees from AstraZeneca KK, Chugai Pharmaceutical Co. Ltd., EP‐SOGO, Novartis Pharma KK, Eli Lilly Japan KK, Taiho Pharmaceutical, Kyowa Kirin, Bristol Myers Squibb, and Merck Sharp and Dohme, outside the submitted work. Yuichi Tambo report receiving personal fees from AstraZeneca KK, Chugai Pharmaceutical Co. Ltd., Takeda Pharmaceutical Company, Merck Sharp and Dohme and Taiho Pharmaceutical, outside the submitted work. Masaki Kanazu report receiving personal fees from AstraZeneca KK, Chugai Pharmaceutical Co. Ltd., Shionogi Pharmaceutical Co Ltd, Boehringer‐Ingelheim Japan, Eli Lilly Japan KK, Merck Sharp and Dohme, and Ono Pharmaceutical Co. Ltd., outside the submitted work. Yuko Tsuchiya‐Kawano report receiving personal fees from Bristol Myers Squibb, Chugai Pharmaceutical Co, Ono Pharmaceutical Co. Ltd, Kyowa Kirin, and Taiho Pharmaceutical, outside the submitted work. Noriko Yanagitani report receiving personal fees from AstraZeneca KK, Chugai Pharmaceutical Co. Ltd., Bristol Myers Squibb, Eli Lilly Japan KK, Pfizer Japan, Bayer Yakuhin Ltd, Takeda Pharmaceutical Company, and Ono Pharmaceutical Co. Ltd., outside the submitted work. Makoto Nishio report receiving personal fees from Merck Sharp and Dohme, Bristol Myers Squibb, AstraZeneca KK, Chugai Pharmaceutical Co. Ltd., Taiho Pharmaceutical, Novartis Pharma KK, Eli Lilly Japan KK, Pfizer Japan, Daiichi Sankyo Healthcare, Boehringer‐Ingelheim Japan, Merck Serono, Astellas, and Ono Pharmaceutical Co. Ltd., outside the submitted work.

## Data Availability

All data generated or analyzed during this study are included in this published article.
